# Editorial: Management of hemodialysis patients

**DOI:** 10.3389/fmed.2022.1116702

**Published:** 2022-12-20

**Authors:** Dipal M. Patel, Rupesh Raina, Bernard G. Jaar

**Affiliations:** ^1^Division of Nephrology, Department of Medicine, Johns Hopkins University, Baltimore, MD, United States; ^2^Akron General Medical Center, Akron Children's Hospital, Akron, OH, United States

**Keywords:** hemodialysis, end-stage kidney disease, arteriovenous fistula (AVF), mineral bone disease, glycemic variability

Chronic kidney disease (CKD) is a highly prevalent condition affecting >10% of the general population (1). Amongst those who progress to end-stage kidney disease (ESKD), ~2.6 million people worldwide receive kidney replacement therapies such as intermittent hemodialysis (HD) (2). Though HD is life-sustaining, it is also associated with increased morbidity and mortality (3). Patients can experience physical effects of fatigue, pruritis, cramping, and deterioration of bone health, and can also be faced with the psychosocial burden of being dialysis-dependent (4). Nephrologists can attempt to reduce ultrafiltration rates to avoid hypotension or cramping, and can prescribe medications to manage mineral-bone disease, anemia, or mental health conditions. Still, current management options are limited, and further research is needed to identify and target factors predisposing HD patients to these poor outcomes. Here, we highlight seven recent articles pertinent to “*Management of hemodialysis patients*.”

Patients who receive HD through an arteriovenous fistula may experience pain during or after dialysis treatments, which can be related to alterations in extremity perfusion (5). These symptoms have limited treatment options, which mostly focus on vascular interventions to revise the fistula, or lifestyle interventions to reduce risk factors for worsening vascular disease (6). In their research article, “*Assessing the efficacy and safety of Juan Bi Tang for dialysis-related myofascial pain in the fistula arm: Study protocol for a randomized cross-over trial*,” Hsu et al. outline a protocol for randomizing HD patients to use of the Chinese herbal medicine Juan Bi Tang, to determine if this therapeutic strategy could alleviate dialysis-related myofascial pain. The authors note wide use of Juan Bi Tang in treatment of other musculoskeletal disorders, though efficacy in treatment of fistula-related pain has not been explored. The results of this study could provide a more effective strategy for managing access arm pain in HD patients.

Additionally, HD treatments can induce hyperglycemia and hypoglycemia in ESKD patients (7). In fact, diabetic patients on HD with high glycemic variability have been shown to have an increased risk of all-cause mortality (8). The ability to check glucose levels quickly and continuously during HD treatments could therefore be impactful. In “*Flash glucose monitoring to assess glycemic control and variability in hemodialysis patients: The GIOTTO study*”, Mambelli et al. demonstrate the acceptability of flash glucose monitoring in patients on HD. Though further studies are warranted to validate its accuracy and precision in the setting of other comorbidities, the use of flash glucose monitoring in HD patients could provide an additional layer of safety in managing these vulnerable patients.

Patients undergoing HD also commonly experience significant muscle weakness and loss of strength (9). In a study by Kang et al., “*Association between alkaline phosphatase and muscle mass, strength, or physical performance in patients on maintenance hemodialysis*,” the authors found that high alkaline phosphatase levels were associated with poor physical performance in ESKD patients on HD, as assessed by several tests of muscle mass and function including handgrip strength and a 6-min walk test. High alkaline phosphatase levels in HD patients typically represent high-turnover bone disease, and have been shown to associate with increased risk of hospitalization and death (10). Future studies may explore the relationships between alkaline phosphatase, strength, and mortality in this population.

Given the massive amount of data collected and available from cohort studies such as the Dialysis Outcomes and Practice Patterns Study (11), advancements in management of HD patients can be enhanced by machine learning and development of prediction models. Kim et al. report use of treatment data from 63,640 HD sessions to develop a deep learning model to predict intradialytic hypotension in their article, “*Deep learning model for predicting intradialytic hypotension without privacy infringement: A retrospective two-center study*”. In this model, systolic blood pressures and mean arterial pressures obtained from hemodialysis machine data are evaluated over 30 min to predict hypotension events in the following 10 min window. This model would not require additional data or bloodwork from the patient. In contrast, in their study “*Personalized prediction of short-term and long-term PTH changes in maintenance hemodialysis patients*”, Pirklbauer et al. describe a personalized mathematical model of parathyroid gland biology, in which dialysate-to-blood ionized calcium gradients and parathyroid hormone (PTH) values are evaluated during HD treatments to predict short-term and long-term changes in PTH levels. This model would use blood and dialysate samples to guide management of mineral bone disease in HD patients, which is highly prevalent and contributes to significant morbidity. We anticipate that big data and mathematical modeling will continue to enable development of prediction models useful in management of HD patients.

There are also several studies focusing on clinical endpoints in HD patients, such as mortality. Some risk of mortality may be related to HD treatments in themselves. In their study, “*Dialyzer classification and mortality in hemodialysis patients: A 3-year nationwide study cohort*,” Abe et al. report that protein-leaking dialyzers may be associated with a reduction in mortality. Compared to low-flux and high-flux dialyzers, protein-leaking dialyzers in a group of >250,000 patients on HD in Japan were associated with lower mortality (HR 0.95, 95% CI 0.92–0.98), though the exact contributions of β2-microglobulin removal were not evaluated. Further information is needed to clarify relationships between removal of variable dialyzable proteins and outcomes such as mortality (12).

Finally, in the era of an ongoing pandemic, we must also acknowledge that non-dialysis related factors can significantly influence the outcomes of patients with ESKD. A study by Tylicki et al., “*COVID-19 vaccination reduces mortality in patients on maintenance hemodialysis*”, provides even more data to support the use of COVID-19 vaccination in patients on HD to reduce mortality.

In sum, the seven articles presented in this Research Topic, “*Management of hemodialysis patients*”, contribute valuable information on assessing and treating some of the variable aspects of increased morbidity and mortality in ESKD patients on hemodialysis.

## Author contributions

DP drafted the editorial. All authors contributed to editing and finalization of the manuscript.
